# Supplementary material for: exFINDER: identify external communication signals using single-cell transcriptomics data
Source: Nucleic Acids Res. 2023 Apr 7;51(10):e58. doi: 10.1093/nar/gkad262 (PMC10250247; doi:10.1093/nar/gkad262)
Supplement: gkad262_Supplemental_File [file gkad262_supplemental_file.pdf]

Supplementary Materials

exFINDER: identify external communication signals using single-cell transcriptomics data

Changhan He[1], Peijie Zhou[1] and Qing Nie[1,2,*]

[1] Department of Mathematics, University of California, Irvine, Irvine, CA 92697, USA

[2] Department of Cell and Developmental Biology, University of California, Irvine, Irvine, CA 92697, USA

* To whom correspondence should be addressed. qnie@uci.edu

The supplementary materials include:

Supplementary Figures S1 to S8

Supplementary Table S1

which begin next page

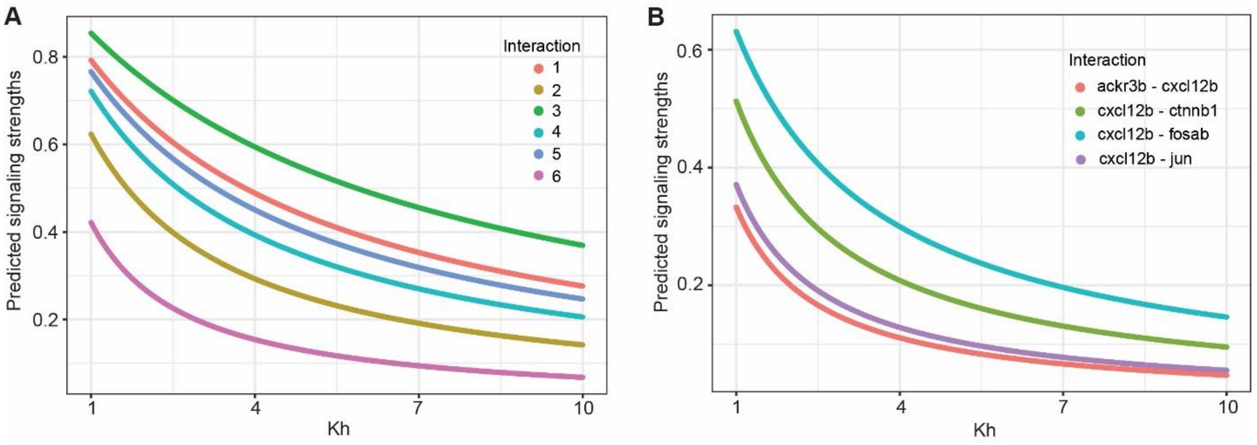

Figure S1. Predicted signaling strengths with different Kh values, showing that changing the value of Kh from 1 to 10 (with step size 0.01) does not significantly affect the relative signaling strengths (i.e. the order of signaling strength) of communication edges. **(A)** Dot plot showing the predicted signaling strengths of the synthetic data. Each interaction contains two genes with random expression values between 0 to 5. **(B)** Dot plot showing the predicted signaling strengths of four zebrafish gene interactions. The corresponding gene expression data is obtained from Tatarakis et al. 2021.

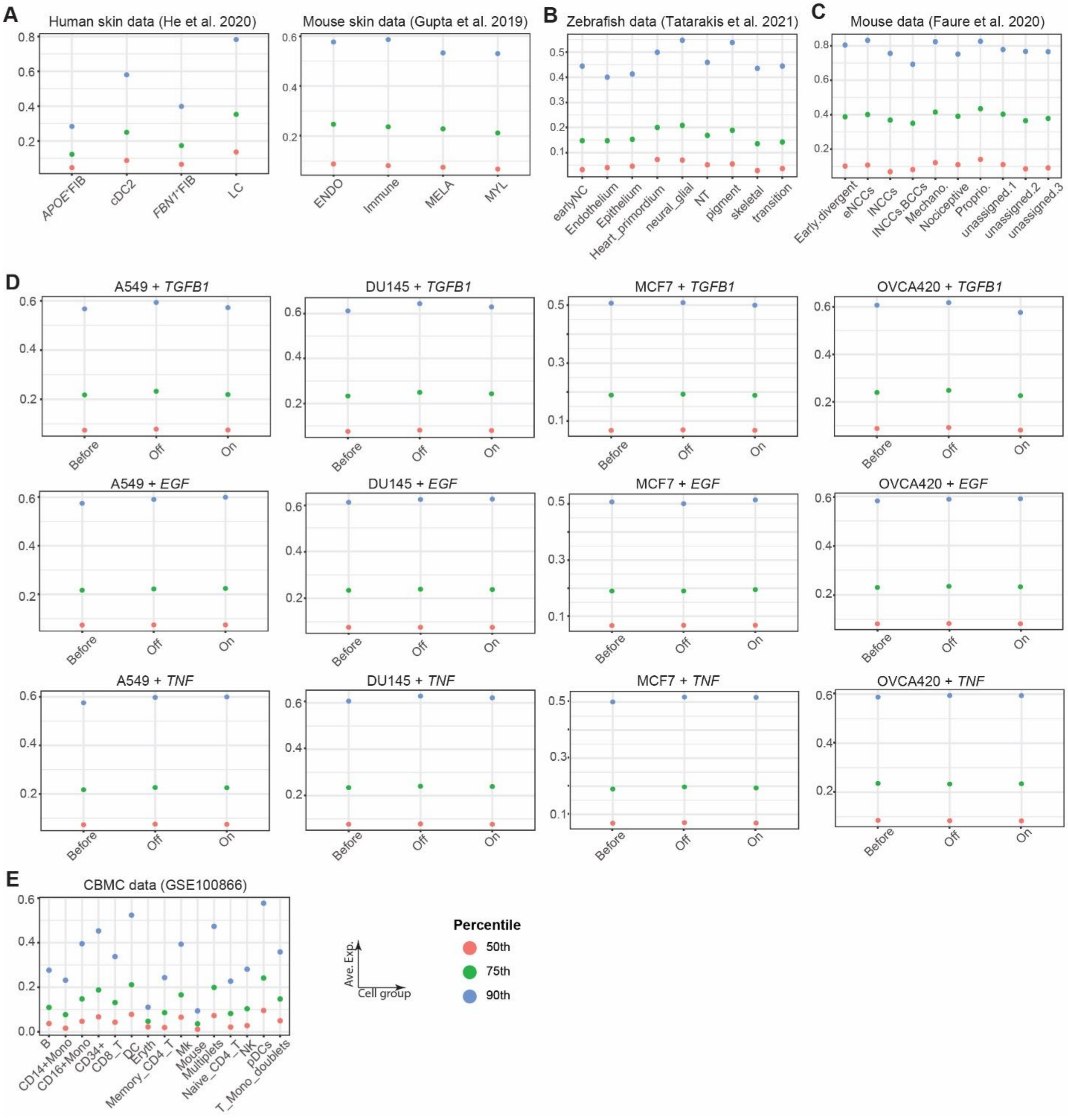

Figure S2. Percentiles for determining cutoff levels for "lowly expressed" and "highly expressed" genes in datasets used. 50th, 75th and 90th percentiles of **(A)** Human skin data (He et al. 2020) and Mouse skin data (Gupta et al. 2019), **(B)** Zebrafish data (Tatarakis et al., 2019), **(C)** Mouse data (Faure et al. 2020), **(D)** A549, DU145, MCF, OVCA420 data (Cook et al. 2020), and **(E)** cord blood mononuclear cells (CBMCs) data (Stoeckius et al. 2017, GSE100866).

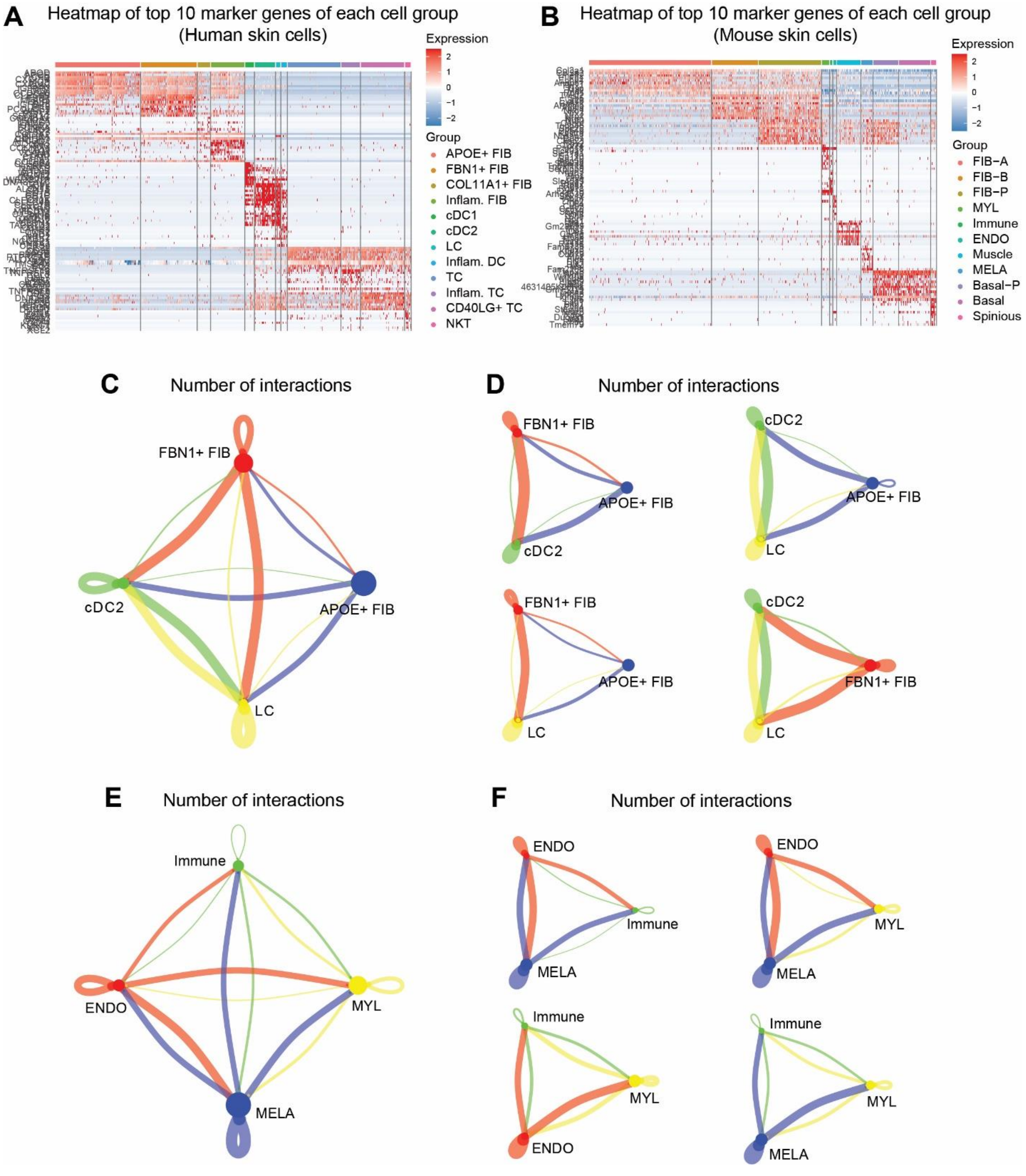

Figure S3. Marker gene expressions and CellChat inferred cell-cell communications in skin datasets. **(A-B)** Heatmap of top 10 marker genes of each human skin and mouse skin cell group (generated by Seurat). **(C-D)** and mouse skin cells **(E-F)** inferred by CellChat. **C, E** Number of interactions between four cell groups. **(D-F)** Number of interactions between every three cell groups.

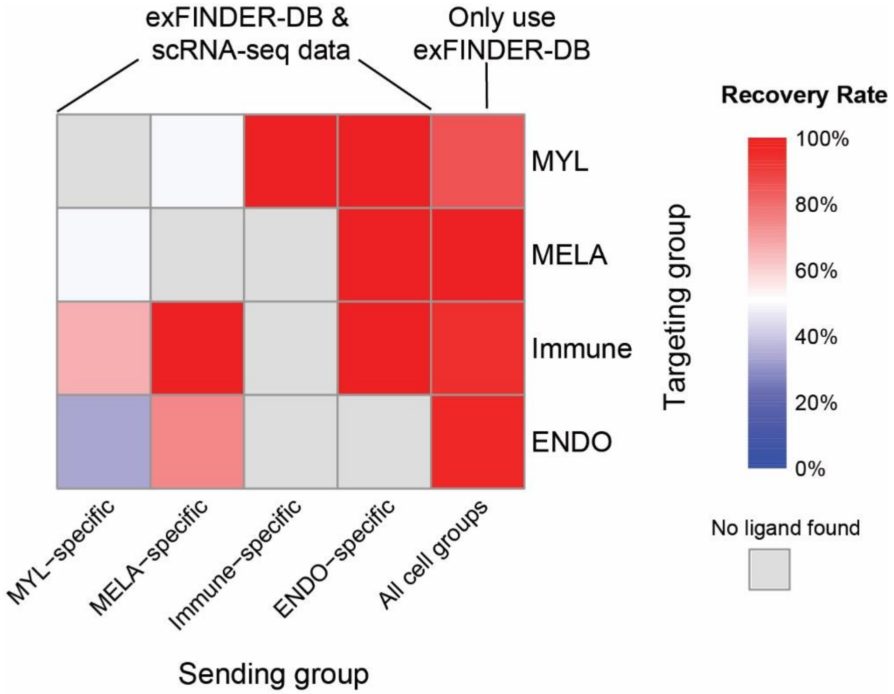

Figure S4. Benchmarking results of exFINDER using dataset of mouse skin cells. Heatmap showing the percentages of ligands inferred by CellChat that also captured by exFINDER. The X-axis represents the cell population groups expressing the ligands, and Y-axis represents the cell population groups receiving the signals. The color bar is the percentages of CellChat-inferred ligands that are also identified by exFINDER. The 5th column shows the ligand recovery rate of exFINDER only using prior knowledge, the rest four columns show the ligand recovery rate of exFINDER using both prior knowledge and the scRNA-seq data. And the grey block indicates no such ligands inferred by CellChat.

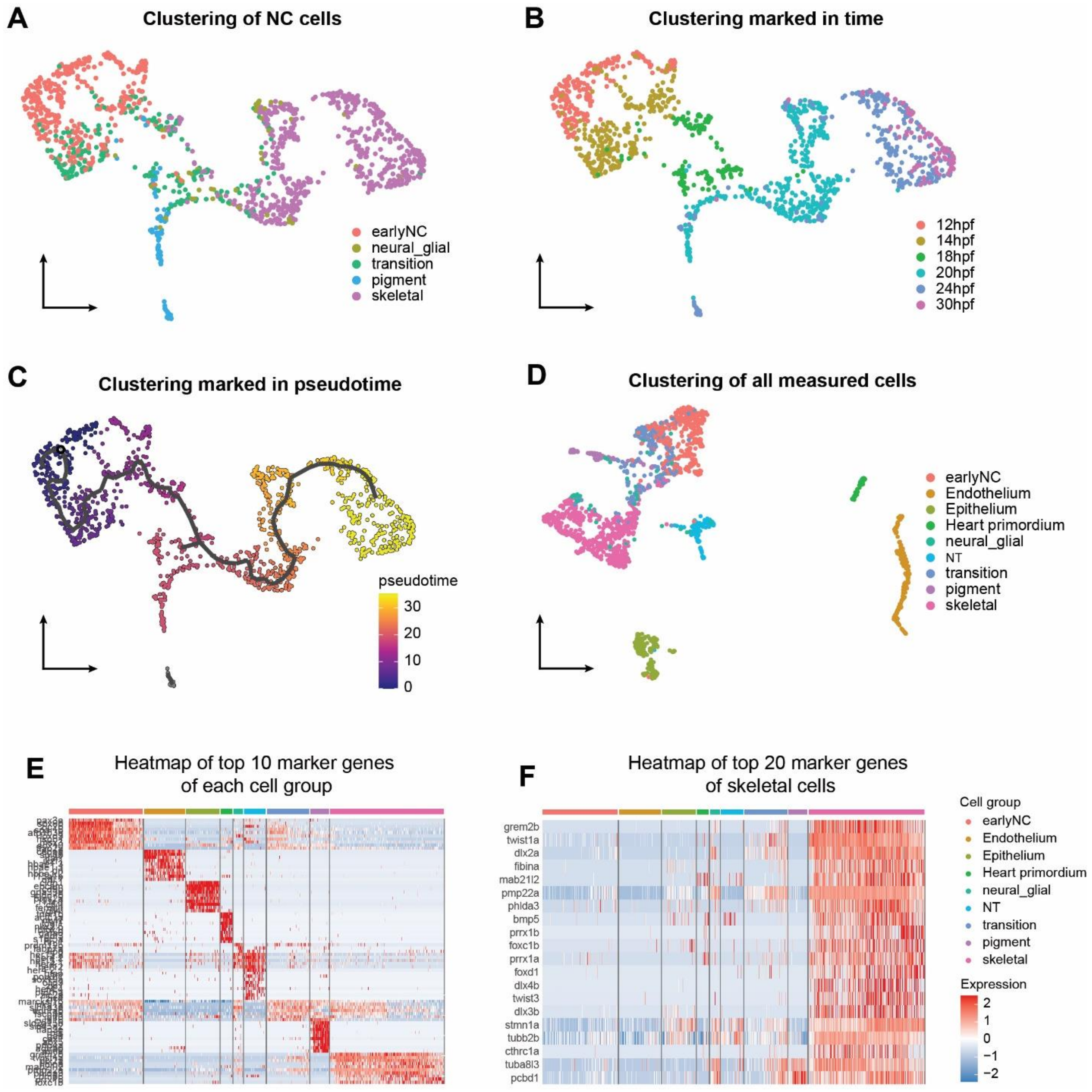

Figure S5. Overview of the dataset of zebrafish neural crest (NC) cells and marker gene expressions (generated by Seurat). **(A)** Reproduction of the dimensionality reduction results of the scRNA-seq data using Seurat. **(B)** Visualization of the dimensionality reduction with time information. **(C)** Trajectory analysis and visualization with pseudotime values using Monocle3. **(D)** Reproduction of the dimensionality reduction results of all measured cells, including NC cells and non-NC cells. **(E)** Heatmap of top 10 marker genes of each cell group. **(F)** Heatmap of top 20 marker genes of skeletal cells.

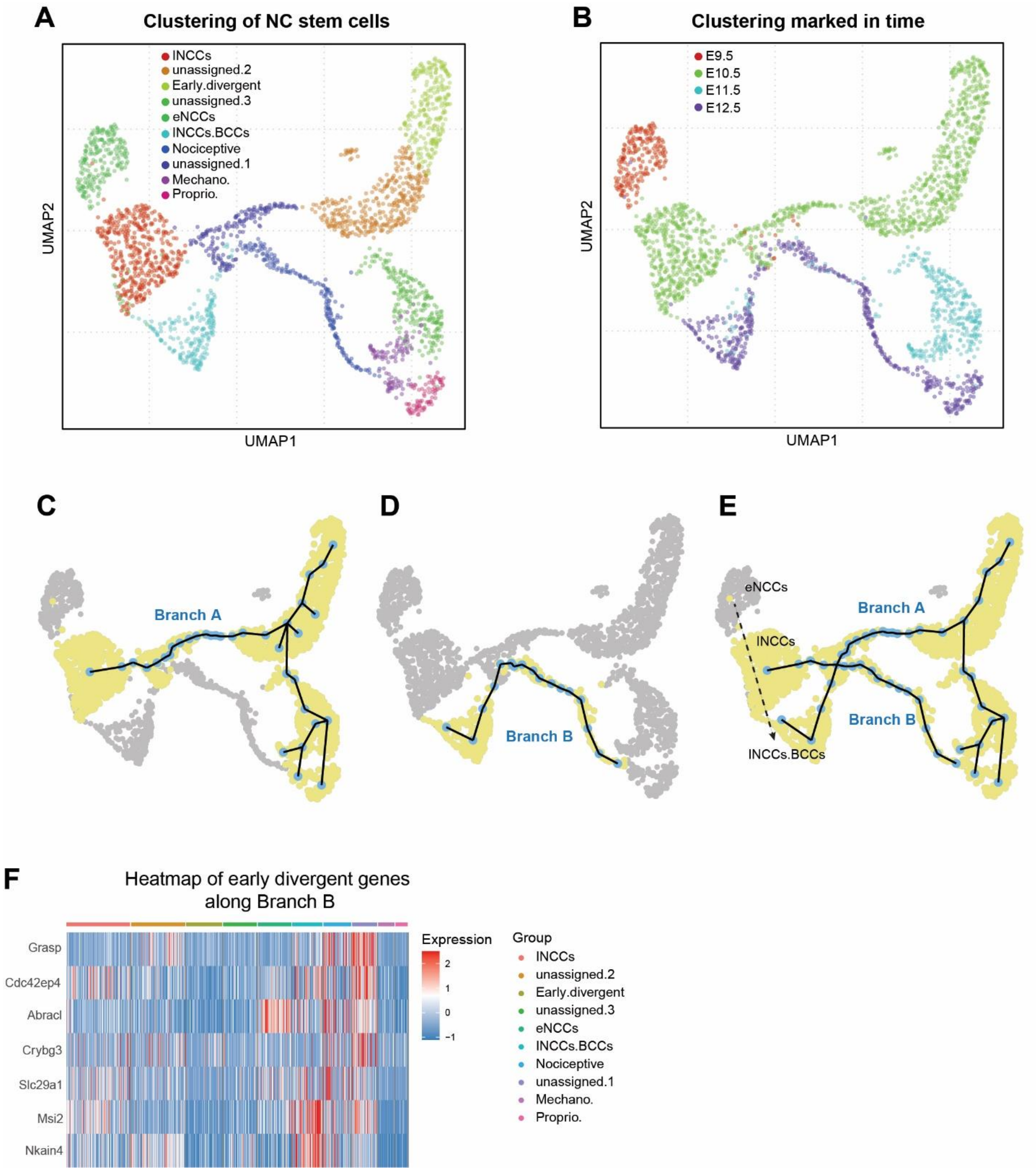

Figure S6. Overview of the dataset of mouse neural crest (NC) cells and marker gene expressions (generated by Seurat). **(A)** Reproduction of the dimensionality reduction results of the scRNA-seq data using pagoda2. **(B)** Visualization of the dimensionality reduction with time information. **(C-E)** Reproduction of the trajectory analysis results using provided codes. **(F)** Heatmap of the early divergent genes along Branch B.

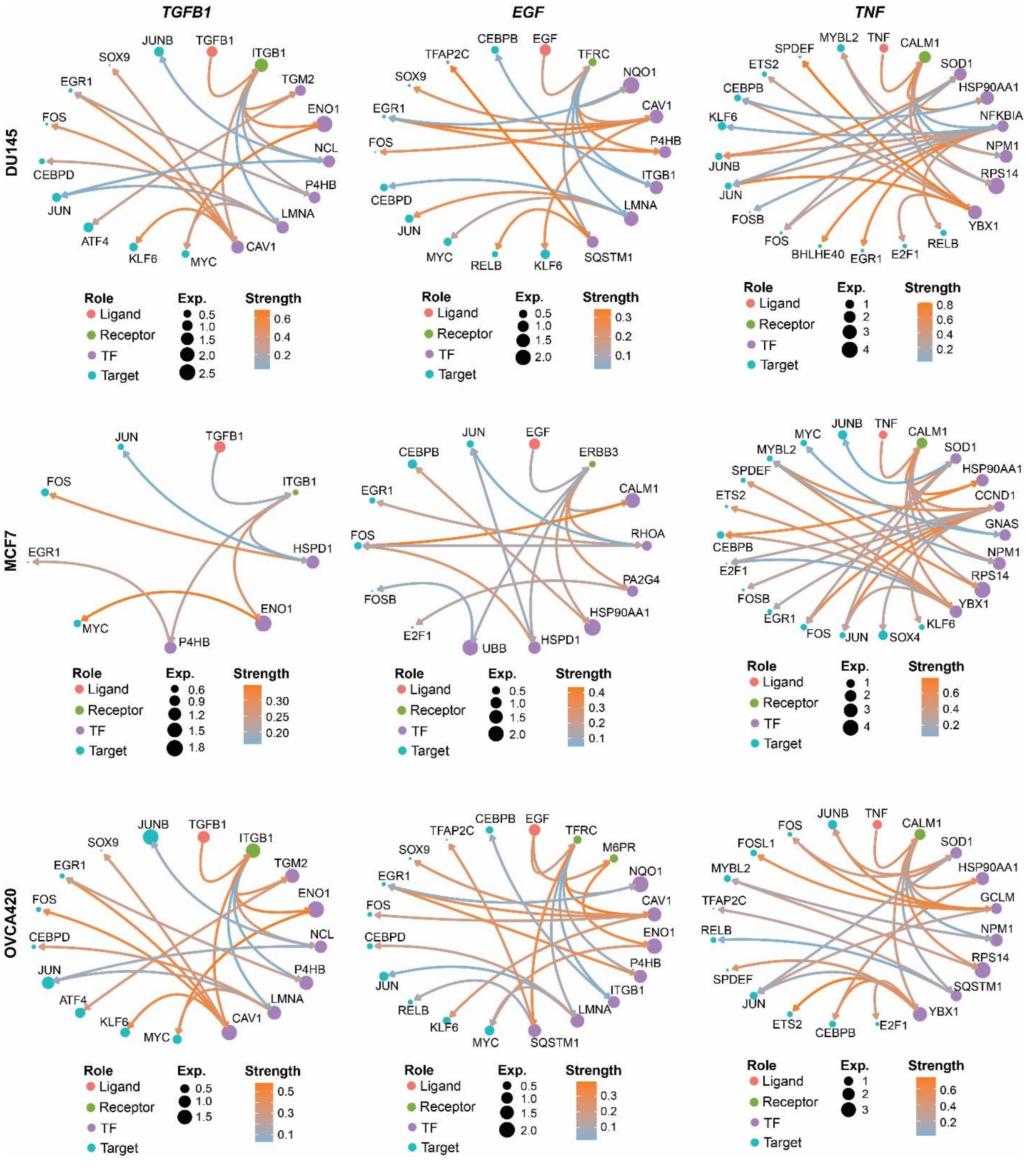

Figure S7. exFINDER inferred inducer-associated exSigNets in EMT datasets. Circle plots showing the inducer-associated exSigNets in DU145, MCF7, and OVCA420 cells.

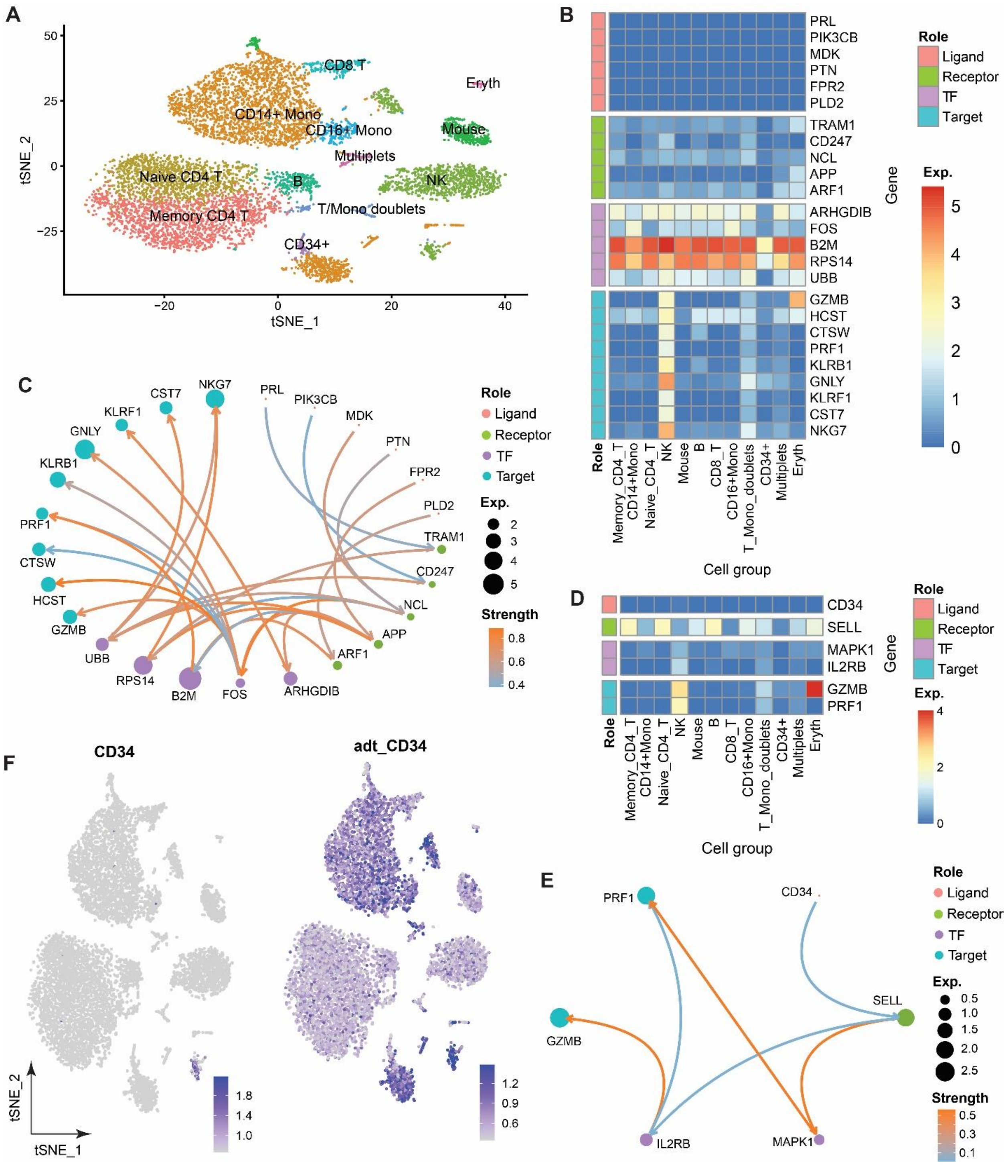

Figure S8. exFINDER analysis using the CITE-seq data of cord blood mononuclear cells (CBMCs). **(A)** Reproduction of the dimensionality reduction results of the CBMCs data using Seurat. **(B-C)** Expression levels and the circle plot of the exSigNet associated with the inferred signals came from the external environment targeting NK cell marker genes. **(D-E)** Expression levels and the circle plot of the exSigNet associated with *CD34* (which did not come from the *CD14*⁺ Mono and *CD16*⁺ Mono cells) targeting NK cell marker genes. **(F)** RNA expression level of *CD34* and its ADT level.

	Number of interactions recorded (human)			Species
	ligand-receptor	receptor-TF	TF-target	
exFINDER-DB	13962	2475457	2963964	Human, Mouse, Zebrafish
NicheNet	12019	2475457	2912482	Human
CellChatDB	2021	None	None	Human, Mouse, Zebrafish
ICELLNET	1034	None	None	Human

Table S1. The number of ligand-receptor, receptor-TF, and TF-target interactions recorded in four different databases, and their availabilities in different species. The number of interactions in a database is calculated based on the interactions with different genes associated with the sending and receiving signals. This quantity is subject to change when a database is updated.
